# Frailty and Adverse Outcomes After Endovascular Revascularization for Chronic Limb-Threatening Ischemia: A Systematic Review and Exploratory Meta-Analysis

**DOI:** 10.7759/cureus.115628

**Published:** 2026-09-01

**Authors:** Mujahed Almomany, Yael Reingold, Ayham Al-Yaghshi, Faisal S Alsayyed, Najim Almajali, Haya Mahmoud, Reeman A Alshyyab, Mahmoud F Jaber, Maya Al-Hijazin, Ahmad Jaber, Ghufran A Al-Shehari

**Affiliations:** 1 General Surgery, Ministry of Health, Amman, JOR; 2 Faculty of Medicine, International University of the Health Sciences, Basseterre, KNA; 3 Faculty of Medicine, Hashemite University, Zarqa, JOR; 4 Faculty of Medicine, University of Jordan, Amman, JOR; 5 Surgery, Internal Medicine, Jordan University Hospital, Amman, JOR; 6 General Practice, Ministry of Health, Amman, JOR; 7 Faculty of Medicine, Jordan University of Science and Technology, Irbid, JOR; 8 Internal Medicine, Jordan University Hospital, Amman, JOR; 9 Faculty of Medicine, Mutah University, Alkarak, JOR

**Keywords:** amputation, chronic limb-threatening ischemia, endovascular revascularization, frailty, peripheral artery disease, prognosis

## Abstract

Frailty is an increasingly recognized prognostic construct in patients with chronic limb-threatening ischemia (CLTI), but its association with adverse outcomes specifically after endovascular revascularization is uncertain. This systematic review and meta-analysis synthesized observational prognostic evidence linking frailty to post-revascularization outcomes in this population. Observational prognostic studies reporting endovascular-specific, frailty-stratified effect estimates in adults with CLTI were included. Frailty was defined using study-specified instruments, including the Claims-based Frailty Indicator, Clinical Frailty Scale, Hospital Frailty Risk score, and Critical Limb Ischemia Frailty Index. Data were extracted into a predefined workbook; risk of bias was appraised with the Quality In Prognosis Studies tool. Effect estimates were analyzed on the logarithmic scale. When pooling was possible, a random-effects model was fitted using restricted maximum likelihood in the metafor package in R. One prioritized contrast per study per outcome group was used to avoid double-counting. When fewer than two independent studies were available for an outcome group, results were summarized descriptively. Three studies were eligible for the main quantitative synthesis, and three additional studies were summarized in narrative or sensitivity analyses. Only one outcome group, the composite of death or major amputation, contained at least two independent studies and was pooled. The exploratory pooled hazard ratio for the prioritized highest/binary frailty contrast versus the lowest/non-frail reference group was 1.81 (95% confidence interval = 0.97-3.39; k = 2; τ² = 0.18; I² = 89.0%; Cochran Q-test p = 0.003 for heterogeneity). This estimate crossed the null. All remaining outcome groups (all-cause mortality, in-hospital mortality, major amputation, readmission mortality, unplanned readmission, and readmission due to major adverse cardiovascular events) were informed by a single study each and were described narratively. Publication bias was not formally assessed because no outcome group reached the prespecified threshold of 10 independent studies. Frailty was generally associated with worse outcomes in individual studies, but pooled evidence was limited to one exploratory outcome and did not reach statistical significance. Standardized, prospective, endovascular-specific studies are needed before firm conclusions can be drawn.

## Introduction and background

Chronic limb-threatening ischemia (CLTI) represents the most advanced stage of peripheral artery disease and carries a substantial burden of amputation and death [1]. Endovascular revascularization has become a common first-line strategy for restoring perfusion, particularly in older patients and those with extensive comorbidity in whom open surgery may be less well tolerated [2]. Even after successful revascularization, outcomes remain heterogeneous, and clinicians need reliable tools to identify patients at the highest risk of adverse events [3].

In older patients with peripheral artery disease, assessment for geriatric syndromes, including frailty, sarcopenia, malnutrition, and mobility impairment, can help identify high-risk patients before revascularization and support safe, goal-concordant care [2]. Frailty reflects reduced physiological reserve and increased vulnerability to stressors [4]. However, frailty is not measured by a single universal instrument. In CLTI, frailty may be captured through non-interchangeable tools, including clinician-rated scales, administrative or claims-based indices, and disease-specific constructs [5-8]. In the evidence base reviewed here, these instruments included the Clinical Frailty Scale, Claims-based Frailty Indicator, Hospital Frailty Risk score, and Critical Limb Ischemia Frailty Index [5-8]. Because these instruments measure overlapping but distinct domains, heterogeneity in frailty measurement was treated as a central limitation when interpreting associations between frailty and post-revascularization outcomes.

A particular challenge is that many available studies combine endovascular and surgical revascularization or report frailty effects for mixed cohorts [8], which limits the interpretability of frailty as a prognostic factor in the specifically endovascular setting. A synthesis restricted to endovascular-specific, frailty-stratified estimates is therefore needed to clarify the direction and magnitude of the association and to characterize the current limits of the evidence. This systematic review and meta-analysis aimed to (1) identify observational prognostic studies reporting endovascular-specific frailty-stratified outcomes in CLTI; (2) quantify the association between frailty and adverse outcomes where sufficient independent studies were available; and (3) appraise the certainty of the available evidence, with explicit attention to heterogeneity in frailty instruments and outcome definitions.

## Review

Methodology

Review Design

The eligibility criteria, outcomes, extraction fields, and analysis rules were defined before quantitative synthesis. The review was conducted and reported in accordance with the Preferred Reporting Items for Systematic Reviews and Meta-Analyses (PRISMA) 2020 statement [9].

Criteria for Considering Studies

All types of observational studies investigating the prognostic role of frailty for adverse outcomes in patients undergoing endovascular revascularization for CLTI were considered. Eligible participants were adults with a confirmed diagnosis of CLTI (guideline-defined, or Rutherford 4-6/Fontaine III-IV) who underwent endovascular revascularization. The prognostic factor of interest was frailty, defined by a validated or clearly specified instrument. The comparator was a non-frail, low-frailty, robust, or lowest-frailty category.

The primary outcomes were all-cause mortality (by time point) and the composite of death or major amputation. Secondary outcomes included in-hospital mortality, readmission mortality, major amputation, major adverse cardiovascular events (MACE), and unplanned readmission.

Search Methods for Identification of Studies

PubMed/MEDLINE, Scopus, Web of Science, and CENTRAL were searched from database inception to June 22, 2026. The search retrieved 642 records: 212 from PubMed, 293 from Scopus, 125 from Web of Science, and 12 from CENTRAL. The PubMed/MEDLINE search strategy was adapted for the other databases. Search terms combined concepts for frailty, CLTI/critical limb ischemia, peripheral arterial disease, and endovascular revascularization. The full search strategy is provided in Appendix A.

Study Selection and Data Management

Records were screened against predefined eligibility criteria, and disagreements were resolved by consensus. Data were extracted into a predefined spreadsheet and checked against the source reports. The following information was extracted: (i) study-related data (year of publication, country, design, data source, sample size, endovascular subgroup, frailty instrument, outcomes, and follow-up); (ii) clinical characteristics of the study populations; and (iii) outcome data as outlined in the Criteria for Considering Studies. The instrument, cutoff, reference category, and outcome availability were extracted separately for each study. The included studies used the Claims-based Frailty Indicator, Clinical Frailty Scale, Hospital Frailty Risk score, and Critical Limb Ischemia Frailty Index.

Assessment of Risk of Bias of Included Studies

The Quality in Prognosis Studies (QUIPS) tool was used to evaluate the risk of bias of selected studies [10]. The tool consists of the following six domains: study participation, study attrition, prognostic factor measurement, outcome measurement, study confounding, and statistical analysis and reporting. The potential risk of bias in each domain was rated as high, moderate, or low. Country and healthcare system were extracted to describe study setting and generalizability, not as independent risk-of-bias domains.

Data Synthesis

When an outcome group contained at least two independent studies, a random-effects model was fitted using restricted maximum likelihood (REML). Outcome groups with fewer than two independent studies were summarized descriptively. Effect estimates were analyzed on the logarithmic scale and back-transformed for presentation. Time-to-event outcomes were summarized as hazard ratios (HRs) and dichotomous outcomes as odds ratios (ORs). HR and OR outcomes were not combined. Different outcomes and timepoints were kept separate. One prioritized contrast per study per outcome group was used to avoid double counting, prioritizing high-versus-low frailty or frail-versus-non-frail over intermediate-versus-low. Heterogeneity was quantified with τ² and I² and the Q-test. Heterogeneity statistics are unstable when the number of studies is very small. Sensitivity and overlapping studies (mixed endovascular/surgical cohorts, combined revascularization cohorts, and overlapping datasets) were excluded from the main pooled analysis and summarized separately. Funnel plots and Egger tests were not performed unless k ≥ 10. Leave-one-out analyses were not performed unless k ≥ 3.

Statistical Software

For data synthesis, the metafor package in R (version 4.6.1; metafor version 5.0-1) was used [11]. Random-effects models were fitted using REML.

Results

Results of the Literature Search

The database search identified 642 records. After removal of 219 duplicate records, 423 records underwent title and abstract screening. Ten reports were sought for retrieval; one report could not be retrieved in full text after database and library access attempts. The remaining nine reports were assessed for eligibility. Three reports were excluded after full-text review: one because it included a mixed open and endovascular cohort without an endovascular-specific frailty effect estimate, one because the comparison was revascularization versus no revascularization rather than frailty, and one because the prognostic model was based on WIfI stage rather than a frailty-stratified estimate. Ultimately, six studies were included in the review: three contributed endovascular-specific estimates to the main quantitative synthesis, and three additional studies were retained for narrative or sensitivity interpretation. The study selection process is shown in Figure 1.

**Figure 1:**
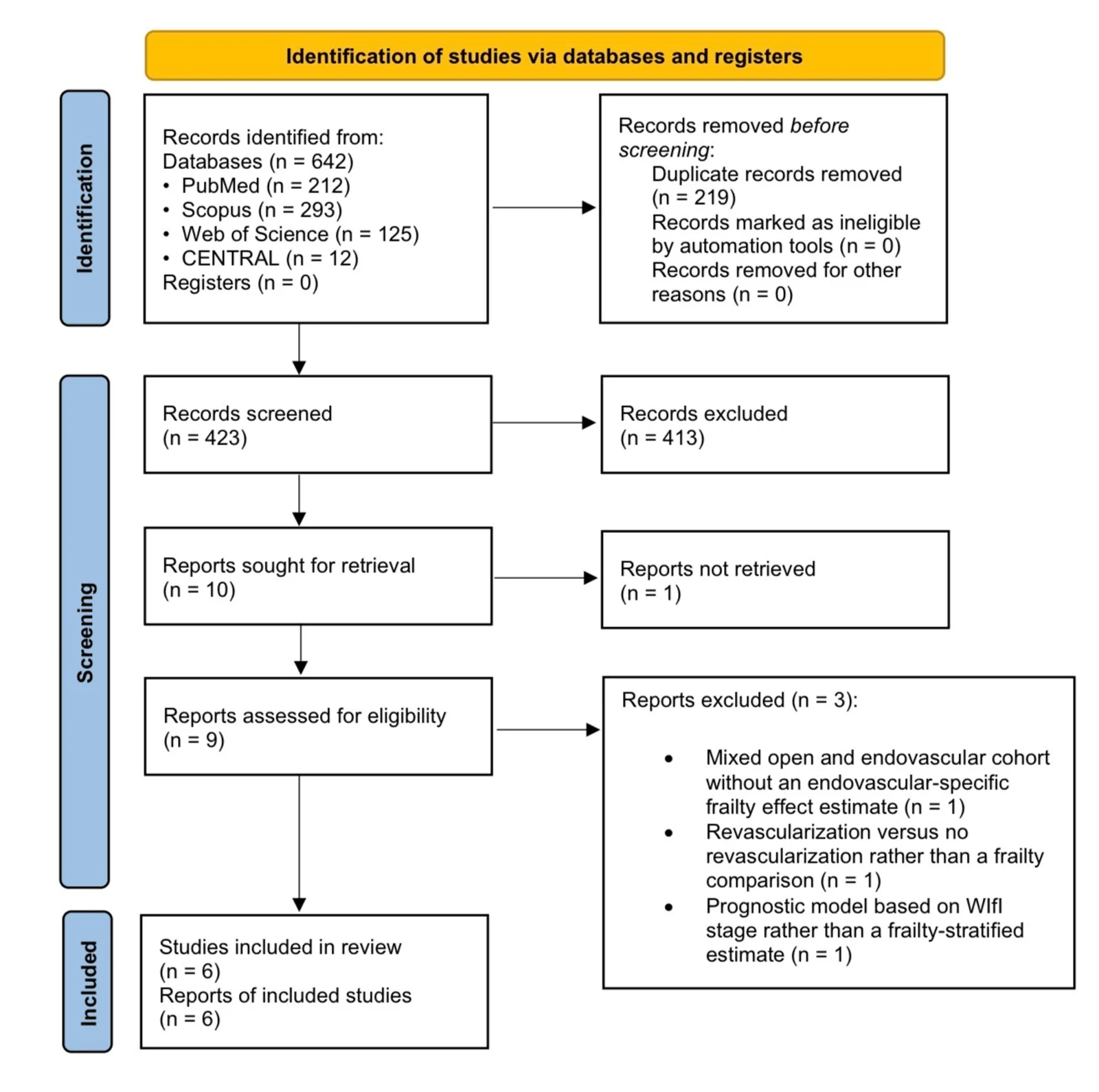
Flow diagram illustrating study selection.

Description of Studies

The three main studies differed in design and data source (Table 1). Butala et al. [12] analyzed a national United States Medicare claims cohort of patients hospitalized with CLTI (N = 85,060 overall), with an endovascular subgroup analyzed using a Claims-based Frailty Indicator and long-term follow-up. Takeji et al. [13] prospectively studied 643 consecutive patients with critical limb ischemia at a single Japanese center, of whom 486 underwent endovascular therapy, using the Clinical Frailty Scale with two-year follow-up. Majmundar et al. [14] analyzed the United States Nationwide Readmissions Database, with an endovascular cohort of 52,935 patients characterized by the Hospital Frailty Risk score, reporting in-hospital and six-month outcomes. The three narrative or sensitivity studies included a mixed endovascular and bypass cohort [8], an infrapopliteal angioplasty cohort in which the frailty construct of interest was not an independent predictor [15], and an end-stage kidney disease cohort in which endovascular and surgical revascularization were reported as a combined revascularization group and therefore could not contribute an endovascular-specific estimate to the primary synthesis [16].

**Table 1:** Characteristics of included studies. CLTI = chronic limb-threatening ischemia; CLI = critical limb ischemia; EVT = endovascular therapy; ER = endovascular revascularization; SR = surgical revascularization; NRD = Nationwide Readmissions Database; ESKD = end-stage kidney disease; NR = not reported; MACE = major adverse cardiovascular events; GNRI = Geriatric Nutritional Risk Index; CLIFI = CLI Frailty Index

Study (year)	Country	Design/Data source	Sample size	Endovascular subgroup	Frailty instrument	Outcomes	Role in synthesis
Butala et al. (2021) [12]	USA	Retrospective cohort; Medicare claims	85,060 (CLTI)	Yes; EVT subgroup (exact n NR)	Claims-based Frailty Indicator	Death/Major amputation composite; long-term follow-up	Main quantitative
Takeji et al. (2018) [13]	Japan	Prospective cohort; single center	643 (EVT 486)	Yes; EVT n = 486	Clinical Frailty Scale [5] (low/intermediate/high)	All-cause mortality; death/major amputation; 2 years	Main quantitative
Majmundar et al. (2023) [14]	USA	Retrospective cohort; NRD	64,338 (ER 52,935; SR 11,403)	Yes; ER n = 52,935	Hospital Frailty Risk score [7] (low/intermediate/high)	In-hospital + 6-month mortality, amputation, readmission, MACE	Main quantitative
Morisaki et al. (2017) [8]	Japan	Retrospective cohort; single center	266 patients (325 limbs)	Mixed EVT + bypass (not separable)	CLI Frailty Index	2-year amputation-free survival	Sensitivity/Narrative
Ruangsetakit et al. (2023) [15]	Thailand	Retrospective cohort; single center	266 patients/286 limbs	Yes; all EVT (infrapopliteal)	CLI Frailty Index	2-year amputation-free survival; CLIFI not independently predictive; GNRI predicted amputation	Narrative
Majmundar et al. (2024) [16]	USA	Retrospective cohort; NRD (ESKD)	49,454 (ER/SR 9,777; NR 39,677)	No; ER/SR combined	Hospital Frailty Risk score [7]	In-hospital + 1-year death/amputation	Sensitivity/Narrative (ER and SR reported as a combined revascularization group)

The included studies used markedly different frailty instruments (Table 2). The main studies applied a Claims-based Frailty Indicator [12], the Clinical Frailty Scale with low, intermediate, and high categories [13], and the administrative Hospital Frailty Risk score with low, intermediate, and high categories [14]. Narrative and sensitivity studies used the Critical Limb Ischemia Frailty Index [8,15] and the Hospital Frailty Risk score in an end-stage kidney disease population [16]. These instruments capture overlapping but distinct constructs: clinically assessed frailty, claims-derived deficits, and hospital administrative risk. This heterogeneity in instruments, categories, and thresholds limits direct comparability of effect estimates and was a central consideration in deciding which outcome groups could be pooled.

**Table 2:** Frailty definitions and outcome availability. HR = hazard ratio; OR = odds ratio; CFI = Claims-based Frailty Indicator; CFS = Clinical Frailty Scale; HFR = Hospital Frailty Risk; CLIFI = CLI Frailty Index; CLI = critical limb ischemia; MACE = major adverse cardiovascular events; EVT = endovascular therapy; ER/SR = combined endovascular/surgical revascularization

Study	Frailty tool	Categories / cutoffs	Reference group	Outcomes available	Effect measure	Synthesis role
Butala et al. (2021) [12]	CFI	Frail: CFI ≥0.25; non-frail: CFI <0.25	Not frail (CFI <0.25)	Death or major amputation (composite)	HR	Main (pooled outcome)
Takeji et al. (2018) [13]	CFS [5]	Low (1–3)/Intermediate (4–6)/High (7–9)	Low (1–3)	All-cause mortality; death or major amputation	HR	Main (pooled + narrative)
Majmundar et al. (2023) [14]	HFR score [7]	Low (≤5)/Intermediate (6–15)/High (>15)	Low (≤5)	In-hospital mortality; 6-month amputation; readmission; readmission mortality; MACE	OR (in-hospital mortality); HR (others)	Main (narrative, single-study outcomes)
Morisaki et al. (2017) [8]	CLIFI	CLIFI positive vs. negative	CLIFI negative	2-year amputation-free survival	HR	Sensitivity (mixed cohort)
Ruangsetakit et al. (2023) [15]	CLIFI	CLIFI positive vs. negative	CLIFI negative	2-year amputation-free survival (not independent predictor)	Not usable for CLIFI	Narrative
Majmundar et al. (2024) [16]	HFR score [7]	Low (<5)/Intermediate (5–10)/High (>10)	Low (<5)	In-hospital death; death or amputation	OR	Sensitivity (ER/SR combined; overlaps 2023 [14])

Risk of Bias of Included Studies

Across the five formally appraised studies, the overall risk of bias ranged from low to moderate (Figure 2). The prospective single-center study [13] was rated at low risk across domains. The large administrative studies [14,16] were rated at moderate overall risk, principally because administrative frailty scores may not capture physiologic frailty, disease severity classifications were not available, and out-of-hospital events could be missed. The claims-based Medicare study [12] was rated at low-to-moderate risk. The mixed-revascularization study contributing only to sensitivity analysis [8] was rated at moderate risk because its frailty estimate was derived from a whole cohort rather than an endovascular-specific subgroup.

**Figure 2:**
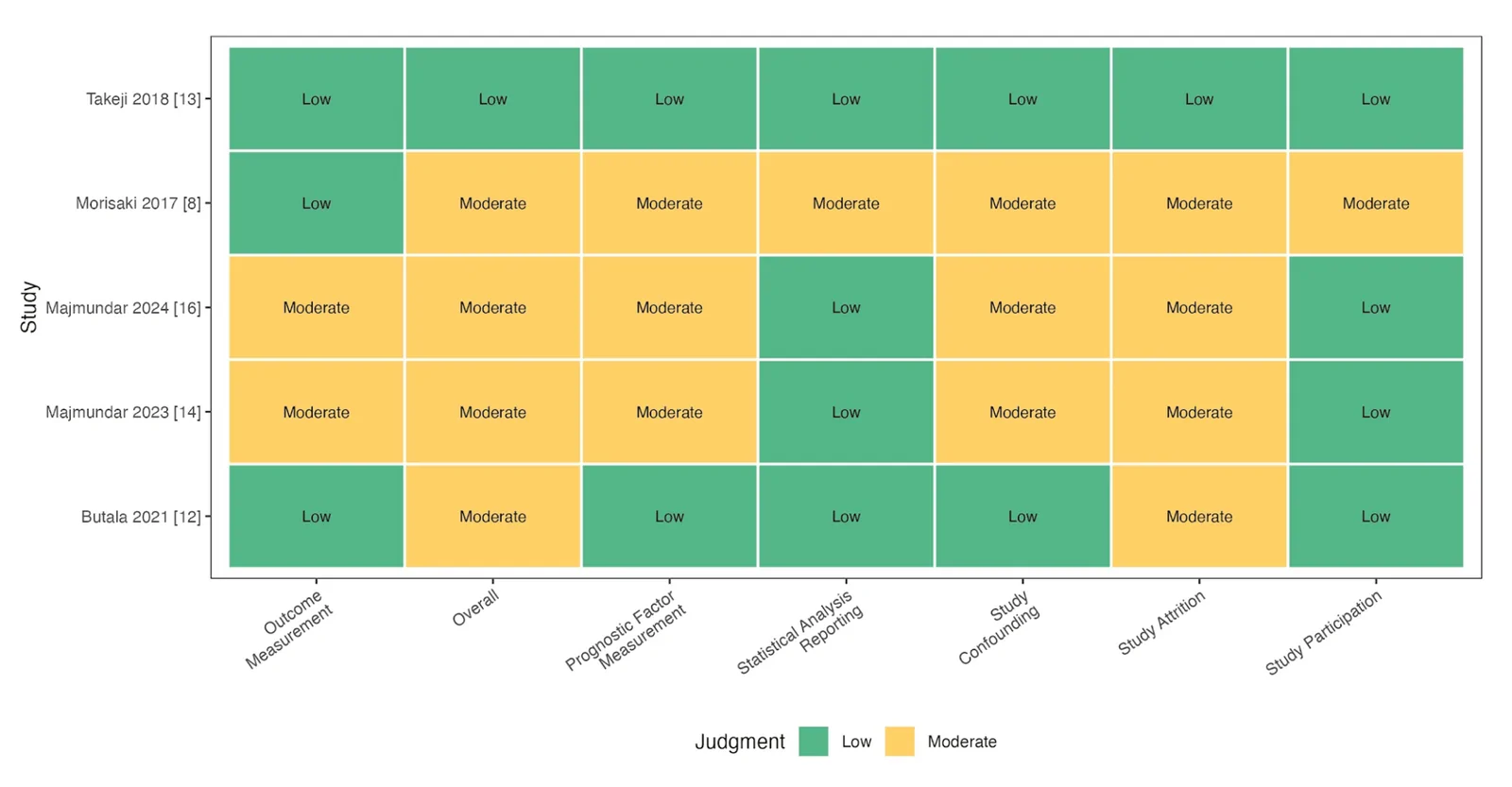
Risk-of-bias assessment using the Quality In Prognosis Studies (QUIPS) tool.

Five studies contributing usable frailty effect estimates to the quantitative or sensitivity synthesis were formally assessed using QUIPS. Ruangsetakit et al. [15] was retained for narrative contextualization because the Critical Limb Ischemia Frailty Index was not available as a usable independent effect estimate and was therefore not incorporated into the formal effect synthesis.

Effects of Prognostic Factor

Only one outcome group, the composite of death or major amputation, contained at least two independent studies and was therefore pooled; the remaining six outcome groups were informed by a single study each and could not be pooled (Table 3).

**Table 3:** Main quantitative, narrative, and sensitivity effect estimates. The pooled outcome is listed first. All other outcome groups were informed by a single study and are descriptive. Random-effects model fitted by REML. Rows represent effect estimates rather than unique studies. Some studies reported more than one frailty contrast or outcome and are therefore displayed in separate rows for transparency. Only one prioritized contrast per study per outcome group was used for any pooled analysis; additional contrasts were summarized descriptively only. HR = hazard ratio; OR = odds ratio; CI = confidence interval; k = number of independent studies; τ² = between-study variance; I² = percentage of variability attributable to heterogeneity; MACE = major adverse cardiovascular events; CLIFI = CLI Frailty Index; REML = restricted maximum likelihood

Study	Outcome	Timepoint	Frailty comparison	Effect measure	Estimate (95% CI)	Synthesis type
Butala et al. (2021) [12] + Takeji et al. (2018) [13]	Death or major amputation	Long-term/2 years	Prioritized highest or binary frailty vs. lowest/non-frail	HR	1.81 (0.97–3.39); k = 2; I² = 89.0%; τ² = 0.18; Q-test p-value = 0.003	Pooled, exploratory
Takeji et al. (2018) [13]	All-cause mortality	2 years	High vs. low	HR	2.32 (1.50–3.59)	Single study, narrative
Takeji et al. (2018) [13]	All-cause mortality	2 years	Intermediate vs. low	HR	1.73 (1.12–2.68)	Single study, narrative
Majmundar et al. (2023) [14]	In-hospital mortality	In-hospital	High vs. low	OR	7.20 (4.40–11.60)	Single study, narrative
Majmundar et al. (2023) [14]	In-hospital mortality	In-hospital	Intermediate vs. low	OR	4.20 (3.20–5.50)	Single study, narrative
Majmundar et al. (2023) [14]	Major amputation	6 months	High vs. low	HR	1.58 (1.45–1.72)	Single study, narrative
Majmundar et al. (2023) [14]	Major amputation	6 months	Intermediate vs. low	HR	1.29 (1.23–1.35)	Single study, narrative
Majmundar et al. (2023) [14]	Readmission mortality	6 months	High vs. low	HR	2.10 (1.78–2.49)	Single study, narrative
Majmundar et al. (2023) [14]	Readmission mortality	6 months	Intermediate vs. low	HR	1.81 (1.61–2.02)	Single study, narrative
Majmundar et al. (2023) [14]	Unplanned readmission	6 months	High vs. low	HR	1.36 (1.29–1.44)	Single study, narrative
Majmundar et al. (2023) [14]	Unplanned readmission	6 months	Intermediate vs. low	HR	1.30 (1.27–1.35)	Single study, narrative
Majmundar et al. (2023) [14]	Readmission due to MACE	6 months	High vs. low	HR	1.65 (1.45–1.89)	Single study, narrative
Majmundar et al. (2023) [14]	Readmission due to MACE	6 months	Intermediate vs. low	HR	1.45 (1.33–1.58)	Single study, narrative
Morisaki et al. (2017) [8]	Amputation-free survival	2 years	CLIFI+ vs. CLIFI−	HR	2.77 (1.78–4.32)	Sensitivity only
Majmundar et al. (2024) [16]	Death or amputation	In-hospital	High vs. low	OR	2.40 (1.90–3.10)	Sensitivity only
Majmundar et al. (2024) [16]	In-hospital mortality	In-hospital	High vs. low	OR	2.70 (1.60–4.80)	Sensitivity only

Primary Outcome

In the pooled random-effects analysis of the death or major amputation composite, using one prioritized contrast per study, the pooled HR was 1.81 (95% confidence interval (CI) = 0.97 to 3.39; k = 2; τ² = 0.18; I² = 89.0%; Q-test p-value = 0.003) (Figure 3). This estimate was designated exploratory because fewer than three independent studies were available, and its CI crossed the null value of 1.0. The high I² is consistent with the differing frailty instruments and contrast structures of the two contributing studies.

**Figure 3:**
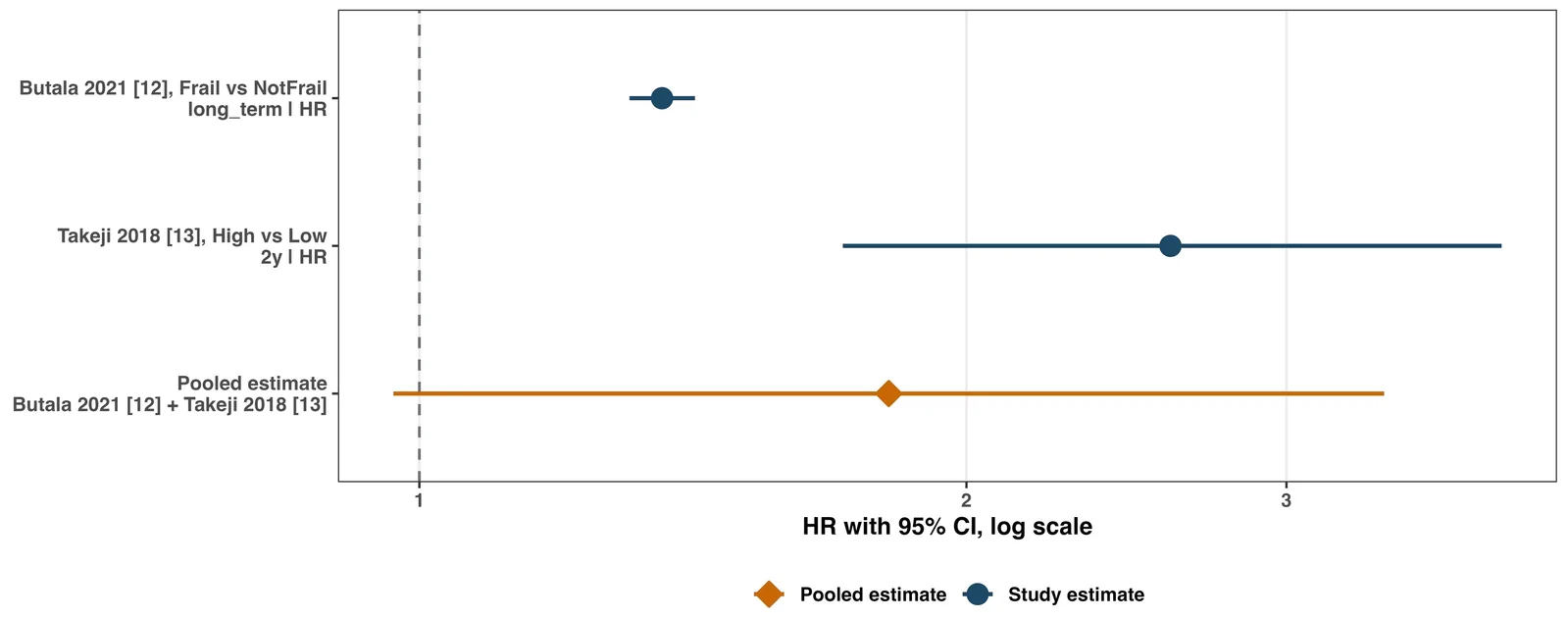
Forest plot of the pooled death or major amputation composite outcome after endovascular revascularization. Effect measure: hazard ratio (HR). Random-effects model using restricted maximum likelihood (REML). Two independent endovascular-specific estimates were included: Butala et al. (2021) [12], frail versus non-frail, adjusted HR 1.36 (95% confidence interval (CI) = 1.31–1.42), and Takeji et al. (2018) [13], high versus low frailty, adjusted HR 2.59 (95% CI = 1.71–3.94). One prioritized contrast per study was used.

Secondary Outcomes

The six single-study outcome groups were summarized descriptively (Table 3; Figure 4). For all-cause mortality at two years [13], the endovascular-specific adjusted HR was 2.32 (95% CI = 1.50 to 3.59) for high versus low frailty and 1.73 (95% CI = 1.12 to 2.68) for intermediate versus low. For in-hospital mortality [14], reported as an OR and kept separate from HR outcomes, the OR was 7.20 (95% CI = 4.40 to 11.60) for high versus low frailty and 4.20 (95% CI = 3.20 to 5.50) for intermediate versus low. For six-month major amputation [14], the HR was 1.58 (95% CI = 1.45 to 1.72) for high versus low and 1.29 (95% CI = 1.23 to 1.35) for intermediate versus low. For readmission mortality, the HR was 2.10 (95% CI = 1.78 to 2.49) for high versus low and 1.81 (95% CI = 1.61 to 2.02) for intermediate versus low; for unplanned readmission, the HR was 1.36 (95% CI = 1.29 to 1.44) for high versus low and 1.30 (95% CI = 1.27 to 1.35) for intermediate versus low; and for readmission due to MACE, the HR was 1.65 (95% CI = 1.45 to 1.89) for high versus low and 1.45 (95% CI = 1.33 to 1.58) for intermediate versus low (all Majmundar et al. [14]). These outcomes should be interpreted descriptively because each was informed by one study.

**Figure 4:**
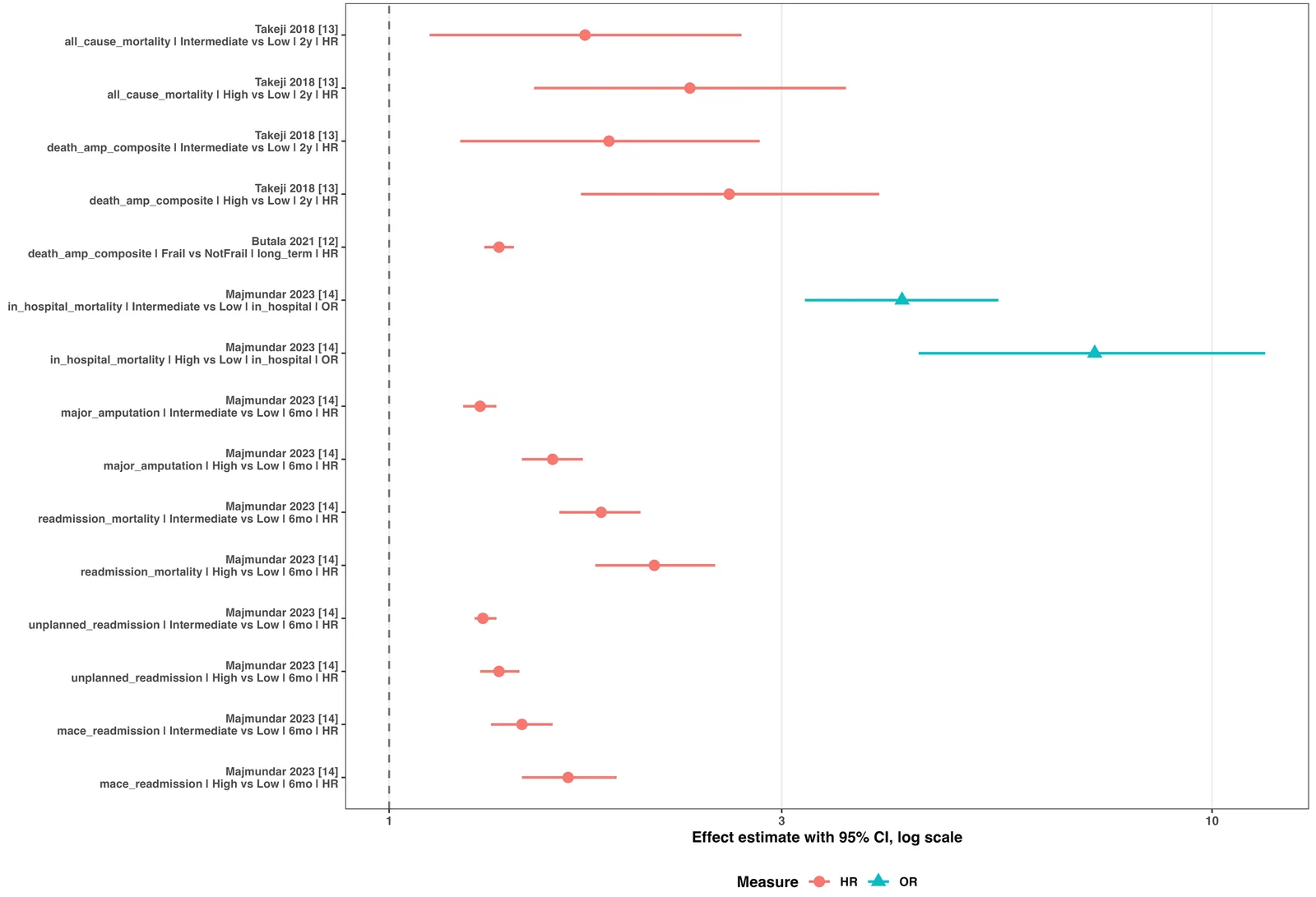
Descriptive summary of extracted main effect estimates across endovascular-specific outcome groups. Hazard ratios (HRs) and odds ratios (ORs) are displayed on a log scale for visual summary only and were not pooled together. Rows represent individual effect estimates rather than unique studies; therefore, studies reporting multiple frailty contrasts or outcomes may appear more than once. Only one prioritized contrast per study per outcome group was used for pooling.

Sensitivity Analysis

Studies excluded from the main pooling were examined separately and are presented as descriptive sensitivity results only (Table 3). In a mixed endovascular and bypass cohort, the Critical Limb Ischemia Frailty Index was associated with two-year amputation-free survival with an HR of 2.77 (95% CI = 1.78 to 4.32) [8]; this estimate reflects the whole revascularized cohort. In an end-stage kidney disease cohort in which endovascular and surgical revascularization were reported as a combined group, the in-hospital ORs for high versus low frailty were 2.70 (95% CI = 1.60 to 4.80) for death and 2.40 (95% CI = 1.90 to 3.10) for death or major amputation [16]. These sensitivity estimates were not combined with the primary analysis because endovascular and surgical revascularization were not reported separately.

Publication bias could not be reliably assessed because no outcome group included at least 10 independent studies; funnel plots and Egger tests were therefore not performed. In the narrative certainty assessment, certainty was judged low for the pooled outcome and low for single-study outcomes, owing to imprecision, indirectness, and inconsistency where applicable (Table 4).

**Table 4:** Narrative certainty assessment for key outcome groups. Certainty was rated cautiously given few studies, diverse frailty instruments, and predominantly single-study outcomes. Formal GRADE was not completed. k indicates number of independent studies. EVT = endovascular therapy; ER = endovascular revascularization; CI = confidence interval; OR = odds ratio; HR = hazard ratio; I² = percentage of variability attributable to heterogeneity

Outcome	k	Studies/Participants	Certainty	Main reasons for caution
Death or major amputation (composite)	2	Butala et al. [12], Takeji et al. [13]; EVT subgroups	Low	Indirectness (different instruments/contrasts); imprecision (CI crosses null); inconsistency (I² = 89%)
All-cause mortality	1	Takeji et al. [13]; 486 (EVT)	Low	Single study; imprecision
In-hospital mortality	1	Majmundar et al. [14]; 52,935 (ER)	Low	Single study; administrative data; OR not HR
Major amputation (6 months)	1	Majmundar et al. [14]; 52,935 (ER)	Low	Single study

Discussion

The main finding of this review with exploratory meta-analysis is that only one outcome, the composite of death or major amputation, could be pooled from two independent studies. The pooled HR was 1.81, but the CI crossed the null (0.97 to 3.39), and the statistical heterogeneity was substantial (I² = 89.0%). Secondary outcomes could not be pooled because each was informed by only one study. While point estimates from individual studies generally suggested worse outcomes among frail patients, single-study data cannot support firm conclusions. The small number of eligible studies reflects the narrow clinical and methodological question rather than absence of broader frailty literature. Many potentially relevant studies combined endovascular and surgical revascularization, included mixed peripheral artery disease or diabetic foot cohorts, did not provide endovascular-specific frailty-stratified estimates, or reported frailty only as a baseline characteristic rather than as a prognostic exposure [8,15-18]. Therefore, the limited number of included studies is a direct consequence of requiring CLTI-specific, endovascular-specific, frailty-stratified effect estimates.

The diversity of frailty instruments claims-based indicators, the Clinical Frailty Scale, the Hospital Frailty Risk score, and the Critical Limb Ischemia Frailty Index makes it difficult to translate the pooled estimate into clinical practice [8,12-16]. CLTI-related immobility may also confound tools that rely heavily on functional status, leading to potential misclassification across cohorts [8,15]. The certainty of the evidence was judged low because most included studies were retrospective. Large administrative datasets provided precise estimates but lacked physiological frailty data and out-of-hospital event capture, while smaller single-center cohorts offered more granular frailty assessments but limited sample sizes [12-14,16]. The pooled outcome was imprecise and inconsistent, and the secondary outcomes were each derived from a single study, making consistency assessment impossible.

A prior systematic review examined frailty across CLTI and diabetic foot populations [17], and another synthesized frailty outcomes in broader vascular surgery cohorts [18]. The present review is more narrowly focused on endovascular-specific, frailty-stratified effect estimates. This narrowed scope made the review more procedure-specific but reduced the number of poolable studies. The included studies are directionally consistent with the existing broader literature linking frailty to adverse outcomes in vascular patients.

Clinically, frailty assessment should not be used as a stand-alone reason to deny revascularization. Consistent with the 2024 American College of Cardiology/American Heart Association peripheral artery disease guideline, assessment of geriatric syndromes, including frailty, sarcopenia, malnutrition, and mobility impairment, may help identify high-risk older patients with peripheral artery disease before revascularization and support safe, goal-concordant care [2]. In the endovascular CLTI setting reviewed here, frailty assessment may also support shared decision-making, patient counseling, postoperative surveillance, rehabilitation planning, nutritional or geriatric referral, and resource allocation. These implications remain provisional because no consensus frailty instrument has been validated specifically for endovascular CLTI populations.

## Conclusions

The available data suggest a directionally consistent association between frailty and worse outcomes after endovascular revascularization for CLTI in individual studies, but the quantitative synthesis was limited to one exploratory pooled estimate for the composite of death or major amputation, and that estimate did not reach statistical significance. This uncertainty arises from the small number of eligible studies, the diversity of frailty instruments and thresholds, heterogeneous outcome definitions and follow-up periods, and the predominance of single-study outcome groups. The strict endovascular-specific eligibility criteria improved interpretability but reduced statistical power, and several potentially relevant studies could not be included because they combined procedures or lacked frailty-stratified estimates. Therefore, these findings should be interpreted as hypothesis-generating rather than definitive evidence of an independent prognostic association. Major gaps remain, including the lack of a validated consensus frailty tool for this specific population, inconsistent reporting of frailty-stratified adjusted estimates, and the confounding effect of CLTI-related immobility on frailty classification. Future research should focus on prospective, endovascular-specific cohorts using standardized frailty instruments, harmonized outcome definitions, and adjusted estimates reported separately from surgical groups to enable more robust meta-analyses. Until such evidence is available, frailty assessment may support risk stratification and shared decision-making, but it should not be used alone to make firm treatment recommendations.

## Appendices

Appendix A: Search strategy

The detailed electronic search strategy is provided below to supplement the brief search description in the Methods section. The PubMed/MEDLINE strategy was used as the base strategy and was adapted for Scopus, Web of Science, and CENTRAL according to each database’s search interface and syntax.

**Table 5:** Search strategy across databases.

Search component	Search terms
Frailty	“Frailty”[Mesh] OR “Frail Elderly”[Mesh] OR frailty[tiab] OR frail[tiab] OR “Clinical Frailty Scale”[tiab] OR “Hospital Frailty Risk Score”[tiab] OR “modified frailty index”[tiab] OR “claims-based frailty indicator”[tiab] OR “CLI Frailty Index”[tiab] OR CLIFI[tiab]
CLTI/Peripheral artery disease	“Peripheral Arterial Disease”[Mesh] OR “Ischemia”[Mesh] OR “chronic limb-threatening ischemia”[tiab] OR “critical limb ischemia”[tiab] OR CLTI[tiab] OR CLI[tiab]
Endovascular treatment	“Endovascular Procedures”[Mesh] OR “Angioplasty”[Mesh] OR “Stents”[Mesh] OR “Atherectomy”[Mesh] OR endovascular[tiab] OR EVT[tiab] OR ER[tiab] OR revascularization[tiab] OR revascularisation[tiab] OR angioplasty[tiab] OR atherectomy[tiab]
Outcomes/Prognosis	“Mortality”[Mesh] OR “Amputation”[Mesh] OR “Patient Readmission”[Mesh] OR “Survival Rate”[Mesh] OR mortality[tiab] OR death[tiab] OR amputation[tiab] OR “amputation-free survival”[tiab] OR readmission[tiab] OR MACE[tiab] OR MALE[tiab] OR “adverse outcome”[tiab] OR prognosis[tiab] OR prognostic[tiab]
Full Boolean strategy	(“Frailty”[Mesh] OR “Frail Elderly”[Mesh] OR frailty[tiab] OR frail[tiab] OR “Clinical Frailty Scale”[tiab] OR “Hospital Frailty Risk Score”[tiab] OR “modified frailty index”[tiab] OR “claims-based frailty indicator”[tiab] OR “CLI Frailty Index”[tiab] OR CLIFI[tiab]) AND (“Peripheral Arterial Disease”[Mesh] OR “Ischemia”[Mesh] OR “chronic limb-threatening ischemia”[tiab] OR “critical limb ischemia”[tiab] OR CLTI[tiab] OR CLI[tiab]) AND (“Endovascular Procedures”[Mesh] OR “Angioplasty”[Mesh] OR “Stents”[Mesh] OR “Atherectomy”[Mesh] OR endovascular[tiab] OR EVT[tiab] OR ER[tiab] OR revascularization[tiab] OR revascularisation[tiab] OR angioplasty[tiab] OR atherectomy[tiab]) AND (“Mortality”[Mesh] OR “Amputation”[Mesh] OR “Patient Readmission”[Mesh] OR “Survival Rate”[Mesh] OR mortality[tiab] OR death[tiab] OR amputation[tiab] OR “amputation-free survival”[tiab] OR readmission[tiab] OR MACE[tiab] OR MALE[tiab] OR “adverse outcome”[tiab] OR prognosis[tiab] OR prognostic[tiab])
